# Impacts of Household Coal Combustion on Indoor Ultrafine Particles—A Preliminary Case Study and Implication on Exposure Reduction

**DOI:** 10.3390/ijerph19095161

**Published:** 2022-04-24

**Authors:** Zhihan Luo, Ran Xing, Wenxuan Huang, Rui Xiong, Lifan Qin, Yuxuan Ren, Yaojie Li, Xinlei Liu, Yatai Men, Ke Jiang, Yanlin Tian, Guofeng Shen

**Affiliations:** 1 College of Urban and Environmental Sciences, Peking University, Beijing 100871, China; luozh@stu.pku.edu.cn (Z.L.); ranxing@stu.pku.edu.cn (R.X.); 2101213348@stu.pku.edu.cn (W.H.); 2001213385@stu.pku.edu.cn (R.X.); 2001111848@stu.pku.edu.cn (L.Q.); yuxuan.ren@pku.edu.cn (Y.R.); yjli2018@pku.edu.cn (Y.L.); liu_xl@pku.edu.cn (X.L.); 1800013210@pku.edu.cn (Y.M.); 1800013254@pku.edu.cn (K.J.); yanlinert@pku.edu.cn (Y.T.); 2 Collaborative Innovation Center of Atmospheric Environment and Equipment Technology, Jiangsu Key Laboratory of Atmospheric Environment Monitoring and Pollution Control (AEMPC), Nanjing University of Information Science & Technology, Nanjing 210044, China

**Keywords:** ultrafine particles, indoor coal combustion, number concentration, size distribution

## Abstract

Ultrafine particles (UFPs) significantly affect human health and climate. UFPs can be produced largely from the incomplete burning of solid fuels in stoves; however, indoor UFPs are less studied compared to outdoor UFPs, especially in coal-combustion homes. In this study, indoor and outdoor UFP concentrations were measured simultaneously by using a portable instrument, and internal and outdoor source contributions to indoor UFPs were estimated using a statistical approach based on highly temporally resolved data. The total concentrations of indoor UFPs in a rural household with the presence of coal burning were as high as 1.64 × 10^5^ (1.32 × 10^5^–2.09 × 10^5^ as interquartile range) #/cm^3^, which was nearly one order of magnitude higher than that of outdoor UFPs. Indoor UFPs were unimodal, with the greatest abundance of particles in the size range of 31.6–100 nm. The indoor-to-outdoor ratio of UFPs in a rural household was about 6.4 (2.7–16.0), while it was 0.89 (0.88–0.91) in a home without strong internal sources. A dynamic process illustrated that the particle number concentration increased by ~5 times during the coal ignition period. Indoor coal combustion made up to over 80% of indoor UFPs, while in an urban home without coal combustion sources indoors, the outdoor sources may contribute to nearly 90% of indoor UFPs. A high number concentration and a greater number of finer particles in homes with the presence of coal combustion indicated serious health hazards associated with UFP exposure and the necessity for future controls on indoor UFPs.

## 1. Introduction

Ultrafine particles (UFPs, with aerodynamic diameter less than 100nm) can adversely affect human health. It has been documented in some epidemiological studies that the number concentration of UFPs positively correlated with the rate of medicine consultation, and mortalities of respiratory diseases, hypertension, and systemic inflammation [1,2,3,4]. For example, one previous study in five European cities found that a 6-day average rise in 2750 ultrafine particles number/cm3 caused a 9.9% increase in the association between UFPs and respiratory mortality [2]. Another study observed that an increase of 0.97μg/m^3^ UFPs caused an increase of 6.3% in systolic blood pressure, 5.6% in diastolic blood pressure, and 8.5% in the high-sensitivity C-reactive protein [3]. The toxicity and potential health outcomes of UFPs are thought to be greater than those of PM_2.5_ or PM_10_. UFPs have larger specific surface areas, resulting in the high adsorption abilities of harmful toxic substances. UFPs more easily reach deep into lung areas and cause many diseases, including serious respiratory diseases, cardiovascular and cerebrovascular diseases, and lung cancer [1,5,6,7]. Moreover, UFPs also play vital roles in influencing climate change via atmospheric cloud condensation nuclei (CCN) [8,9].

UFPs can be produced in many combustion processes or secondarily from precursors. Globally, vehicle emissions and residential combustions are large sources of UFPs, contributing 40% and 17% of the total for the year 2010 [10]. In developing countries such as China and India, residential combustions are major sources of primary UFPs, and the emissions are expected to continuously grow in some areas, such as India, due to the increase in the activity levels in industrial, traffic, and coke production in India [10]. Several emission measurement studies have pointed out that the size of particles from indoor solid-fuel combustion are very small, with high fractions of UFPs [11,12,13]. However, so far, studies on emission and household air pollution associated with solid-fuel use mostly focus on the bulk PM_2.5_ [14,15,16], with much less information on particle size and UFPs [11,17]. Biomass and coal burning can emit high concentrations of PM_2.5_ in a short duration, contributing to a large ratio of indoor PM_2.5_ and resulting in severe indoor air pollution [18,19]. Available studies associated with UFPs from indoor solid-fuel use, either in emissions or indoor air, are mostly conducted on the use of firewood, while studies on coal combustion are relatively rare [20,21,22,23,24,25,26]. It was reported that particles in coal-combustion emissions were nearly all UFPs [11], and the number concentration and size distribution of UFPs vary among different coal types and burning conditions. Some so-called “clean” coals (produce less PM_2.5_ per unit mass fuel combustion, such as semi-coke coals) may produce more UFPs than the “dirty” ones (produce more PM_2.5_ per unit mass fuel combustion, such as bituminous) [12,27]. A greater number of finer particles were observed in emissions during the stable combustion stage in comparison with the ignition and fierce combustion stages [28].

Coals are an important primary energy in China [29,30]. Approximately two-fifths of population in China, with most in rural areas, still use coal and biomass for heating or cooking [31,32]. Besides stoves without chimneys that directly discharge particulate and gaseous pollutants (including particles, CO, SO_2_, PAH, etc.) into indoor environments, stoves with chimneys can also yield significant amounts of air pollutants indoors, known as indoor fugitive leakages, which lead to severe indoor air pollution [15,16,33]. Few studies have investigated indoor UFPs in coal-use homes. One study in rural Guizhou, southwest China showed that when honeycomb briquettes were burned in home stoves without chimneys for cooking (by boiling a pot of water in experiments), there were high levels of particles with diameters of 60–80 nm, and the particle size distribution was unimodal [24,25]. Another study in rural Hebei, north China reported that indoor UFP number concentrations during the cooking period achieved up to 2.5 × 10^5^ #/cm^3^ in homes burning bituminous coals in chimney stoves [27], which was much lower than the concentrations measured in homes using unvented stoves [24,25]. Available studies on indoor UFPs in rural households in China are still limited, especially those on the indoor–outdoor relationship and quantitative sources of indoor UFPs in rural households. These studies called for greater concern for the severe pollution status of indoor UFPs, especially for those households that have a high reliance on coals in daily life. To study the influence of indoor coal combustion and outdoor air infiltration on the indoor UFP level and size distribution, this study simultaneously measured the particle number concentrations of size-segregated particles in indoor and outdoor environments. The indoor–outdoor relationship and its dynamics changes over the period when coals were combusted indoors were analyzed and discussed. With highly resolved temporal data, this study also estimated the relative contributions of indoor and outdoor sources on indoor UFPs by using a statistical approach.

## 2. Methods

### 2.1. Experimental Description

The field test was performed in rural Shanxi, northern China in February 2021. In the studied area, coals are widely used as the main household energy for daily cooking and heating. Low-quality raw chunks have mostly been banned, and honeycomb briquettes with lower volatile-matter contents are often used. The measurement was conducted in a typical rural household, in which anthracite briquettes were burned for cooking and heating. The coal was a 9-hole cube honeycomb briquette (space between numbers and units 9 cm × 9 cm × 9 cm) that was stacked into 4 layers in a steel chimney stove. The sampling period covered the stage from pre-combustion to ignition to flaming during the heating activity. The indoor sampling inlet was in the middle of the room, ~1.5 m above the ground and about 1.5 m away from the stove, while the outdoor sampling inlet was in the yard and 1.5 m above the ground (Figure S1). During the measurement period in winter, the door and windows were closed to keep warm. As a comparison of the indoor–outdoor relationship and particle size distributions, an indoor measurement using the same measurement was performed in one urban room without combustion sources present. There were also no people present, and the windows were open during the test. The size of this comparison room was about 20 m^2^, and it was located on the top floor of an urban building (Figure S1).

### 2.2. Instrument and Quality Control

To measure particle number concentration and particle size distribution of indoor particles during coal combustion in field conditions, NanoScan SMPS (scanning mobility particle sizer 3910, TSI Incorporated, Shoreview, MN, USA) was used to measure indoor and outdoor UFPs simultaneously [34]. NanoScan SMPS 3910 is a portable particle size spectrometer with a small volume but relatively high measurement accuracy. The instrument measures 13 size channels from 10–420 nm with a high time resolution of 1 min (scan up for 45 s and scan down for 15 s) in Scan Mode and a wide concentration range (100–1,000,000/cm^3^). NanoScan SMPS 3910 uses an Isopropyl Alcohol-based CPC to measure particle number concentrations by counting single particles. Our test concentrations were all in the measuring range. Scan Mode was used. The instrument was calibrated in the laboratory by the manufacturer and overhauled prior to its use in the field. The instrument inlet was cleaned and zero-calibrated before each sampling. In the rural household, indoor PM_2.5_ mass concentration was also simultaneously measured using the Model 8533 Dusttrak DRX monitor (TSI Incorporated, Shoreview, MN, USA) with a resolution of 0.001mg/m^3^. The Model 8533 Dusttrak DRX monitor is a portable real-time aerosol monitor commonly used for detecting particulate matter mass concentrations by light scattering. Its measuring range is 0.001–150 mg/m^3^. It was placed indoors beside the indoor inlet of the SMPS.

### 2.3. Data Analysis

To quantify the relative contribution of indoor UFPs from different sources, firstly, outdoor concentration and infiltration factors were combined to calculate the contribution of outdoor sources [35]. The UFPs from internal sources except coal combustion were derived from indoor concentration during no combustion, subtracting the outdoor contribution. The remaining part was the contribution of coal combustion. Data processing and statistical analysis were carried out using Matlab R2019b (MathWorks, Beijing, China) and the SPSS (IBM SPSS Statistics 26) (IBM, Beijing, China). During the study period, the UFP number concentration measured did not follow a normal distribution nor a log-normal distribution (Figure S2); therefore, non-parametric methods were adopted in the statistical analysis. A significance level of *p <* 0.05 was adopted. A graphical drawing was prepared using Origin 2021b and Excel 2020.

## 3. Result and Discussion

### 3.1. Indoor and Outdoor UFP Number Concentrations

**Figure 1:**
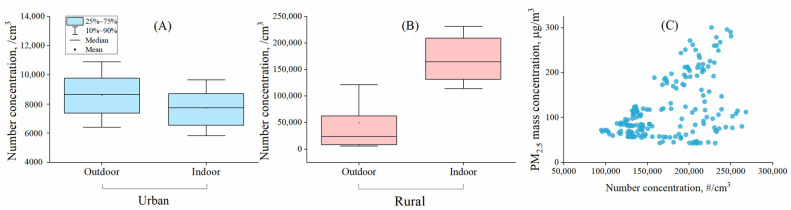
Indoor and outdoor UFP number concentrations in the urban room without internal combustion sources (**A**), the rural household with coal combustion indoors (**B**), and the relationship between indoor UFP number concentration and PM_2.5_ mass concentration in the rural household burning coal (**C**).

In the urban room without internal combustion sources, the indoor UFP number concentration was positively correlated with that outdoors (*p* < 0.001, r = 0.978), but the outdoor level, at a median value of 8660 #/cm^3^, with the interquartile range [IQR]: 7,370–9,770 #/cm^3^, was higher than the indoor level (7740 #/cm^3^, IQR: 6540–8700 #/cm^3^) (*p* < 0.05) by about 10% (Figure 1A). Traffic emissions are believed to be one important source of UFPs in urban areas [36]. Ambient UFP number concentrations would decrease with an increase in the distance from highways/roadside [37]. One previous study reported that the roadside UFP concentration was nearly two times that in the background area, and the median level was 8617 #/cm^3^, which was close to our result [38].

In the rural household with coal combustion indoors, the indoor UFP concentration was as high as 164,300 (IQR:131,560–208,890) #/cm^3^, and was nearly seven times the outdoor UFP concentration (23,520 #/cm^3^, IQR: 8120–61,700 #/cm^3^) (Figure 1B). Generally, the indoor and outdoor UFP number concentrations were also positively correlated (*p* < 0.001, r = 0.490). Even during the period when coals were not combusted, the indoor UFP number concentration was higher than the outdoor, with a difference of about five times. The ratio of indoor and outdoor UFP (I/O) and the change in I/O values are discussed in detail later.

To date, little information is available for indoor UFPs from coal-burning homes. We found three studies that reported indoor UFP number concentrations and/or size distribution when coals were burned for cooking. The study by Zhang et al. (2012) measuring indoor particle number concentrations (10 nm–10 μm) in rural Guizhou, China, reported a very high peak concentration of 1.3 × 10^7^ #/cm^3^ when honeycomb briquettes were burned in an unvented stove for cooking, and after about 2 h, when there were no burning activities indoors, the total particle number concentrations decreased by about 75% [24]. One reason for such high levels in this study was that the stove used in the testing room was unvented, which directly produced emissions into the indoor air. Another study was in rural Hebei, north China, which reported that when bituminous coals were combusted in a chimney stove, the indoor particle (3 nm–10 μm) number concentration was 2.5 × 10^5^ #/cm^3^, and nearly one order of magnitude higher than the background (2.0 × 10^4^ #/cm^3^) [27]. The levels were higher than those of our measurement in the rural household. Besides the different coals and stoves studied, the difference between cooking and heating activities also affected emissions and indoor pollution levels. Indoor heating is a relatively long process compared to cooking, and residents usually keep the coal combustions in heating stoves smoldering with low modified combustion efficiency. For cooking, on the other hadn, the duration is short, and the burning usually occurs in flaming conditions. The peak number concentrations in Wang et al. (2020)’s study were close to our present study (2.6 × 10^5^ #/cm^3^) [27]; however, it decreased quickly by an order of magnitude, to about 2.0 × 10^4^ #/cm^3^ in ~40 min, when the burning was over, while in the present analysis on heating stoves, the indoor UFP number concentration during the smoldering burning period was about half of the peak concentration and lasted for a much longer time.

While it is more likely to form fine particles via condensation and coagulation [27,39], the association between indoor PM_2.5_ and UFPs is not explicit. In the rural household, the median PM_2.5_ was 85.0 μg/m^3^ (IQR: 66.6–142 μg/m^3^), which was nearly six times the new WHO air quality guideline (daily valve: 15 μg/m^3^). There is still no standard on UFPs. PM_2.5_ mass concentration was found to be positively correlated with the UFP number concentration (r = 0.402, *p* < 0.001) (Figure 1C). It was found that, in rural households from Honduras, PM_2.5_ mass concentration and particle number concentration were highly correlated in homes burning wood in traditional stoves (r *=* 0.93) and moderately correlated in homes using improved stoves (r *=* 0.67) [39]. However, it is important to note that, in some emission studies, it was found that some fuel–stove combinations had low PM_2.5_ emissions, but not necessarily low UFPs [11,40]. For example, Wang et al. (2019) found that the UFP emission factors for anthracite (1.7 × 10^16^ #/kg) was higher than bituminous (1.1 × 10^16^ #/kg) [11], and much higher than firewood (0.5 × 10^16^ #/kg) and charcoal (0.8 × 10^16^ #/kg); however, the PM_2.5_ emission factors had a different trend, with the lowest in anthracite and the highest for bituminous. Given the significant adverse impacts on human health of UFPs, it is important to act in controlling UFPs and its primary sources; the control strategy may be different from that for PM_2.5_ as the two do not always exist synchronously in emissions and air.

### 3.2. Distinct UFP Size Distribution

**Figure 2:**
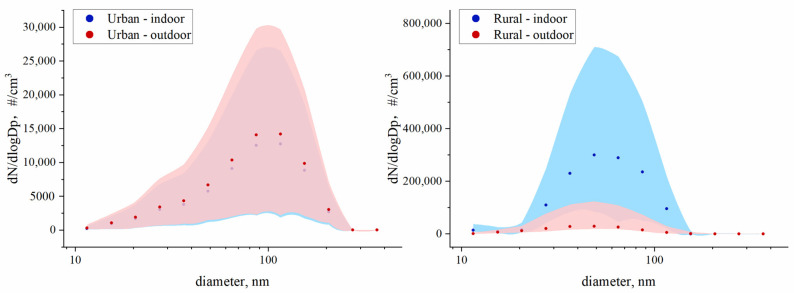
Size distributions of UFPs in indoor and outdoor air from the urban household without indoor combustion sources (**left**) and the rural room burning coals (**right**). Dp means particle diameter.

In both indoor and outdoor air, the UFP size distribution was unimodal. In the urban room without an indoor combustion source, over 70% of the particles were between 56.2 nm and 177.8 nm, and the highest number concentration of 1.3 × 10^4^ #/cm^3^ was for particles of 115.5 nm (Figure 2). The corresponding outdoor UFP size distribution was, again, like that indoors, but the indoor concentration was lower. The urban room without an indoor combustion source was obviously influenced by the nearby traffic emissions, which produced high UFP concentrations of 20–150 nm [32,41].

In the rural household burning coals, the particles with diameters at 31.6-100 nm were much more abundant (~80%), and the particles with 48.7 nm had the highest number concentration of 3.0 × 10^5^ #/cm^3^ at peak. The corresponding outdoor UFP size distribution was similar, with a number concentration peak at 48.7 nm. However, the overall number concentration outdoors was appropriately one order of magnitude lower than that indoors. The size distribution of UFPs in the rural household burning coals was like some, though limited, past studies [24,25,27]. A study during a winter in southwestern China conducted by Zhang et al. (2014) reported that the size of the most abundant particles ranged 40–70 nm in households burning wood and anthracite indoors [25]. Another study in rural Guizhou, southwest China reported a similar result of a unimodal distribution with the most abundant particles at 50–70 nm during the flaming stage of burning honeycomb briquettes made of anthracite coal and clay [24]. For bituminous coals, the most abundant particles were also very fine ones at, for example, 30 nm in a rural household in rural Hebei, northern China [27]. Particle size distribution is affected by a number of factors, such as fuel type, stove difference, combustion temperature, combustion efficiency, etc. [27,28,42,43]. In emission measurements, it was reported that the size of UFPs from the bituminous coal combustion were mostly between 10 nm and 100 nm [12]. High temperature conditions in some forced-draft stoves can yield high fractions of finer and ultrafine particles, even though the total particle number concentrations or emission factors could be lower compared to the burning in relatively low-temperature natural-draft stoves [13,40]. The size distribution characteristics of UFPs in the rural home confirmed significant impacts of coal combustion on indoor air quality. The similar size distributions of UFPs measured in the outdoor yard indicated that the near outdoor air was obviously influenced by the indoor burning activities. In fact, the dynamic analysis of paired indoor–outdoor UFP changes clearly illustrated the impacts of indoor burning on ambient air, which is a part of the “neighborhood effect”.

### 3.3. Ratio of Indoor-to-Outdoor UFP

I/O reflects the relative impacts of indoor and outdoor sources, although it may not be the best indicator of indoor–outdoor exchange compared to parameters such as infiltration and penetration factors [44]. In the rural household with coal combustion, the I/O ratio, on average, was about 6.4 (IQR: 2.7–16.0), indicating significant contributions from the internal sources, e.g., coal combustion in the present study, while in the urban room, the I/O ratio averaged at about 0.89 (IQR: 0.88–0.91), which was close to 1.0 (*p <* 0.001), resulting from a good ventilation condition and the absence of internal sources. There were no studies on the I/O ratio of UFPs in coal-combustion homes, but a few studies on the I/O of UFPs in some urban households or offices. From a study on six urban homes in Hanoi, Quang et al. reported that the I/O ratio for particles of 10–300 nm was 0.66 ± 0.26. Buonanno et al. (2013) reported a similar I/O value of 0.63–0.74 in schools in Italy [45]. When there were indoor sources, such as cooking, smoking, and use of candles, Madureira et al. (2020) showed that the I/O ratio of UFPs was 1.59, ranging from 0.27–6.67, in households in Oporto, Portugal [46]. The I/O ratio varied across different particle size fractions, especially in the rural household with coal combustion. The highest I/O ratio was observed for the finest particle of 10–13.3 nm. This is due to high emissions of fine and ultrafine particles from the coal combustion, which has been documented in many emission studies in the literature [27,28].

**Figure 3:**
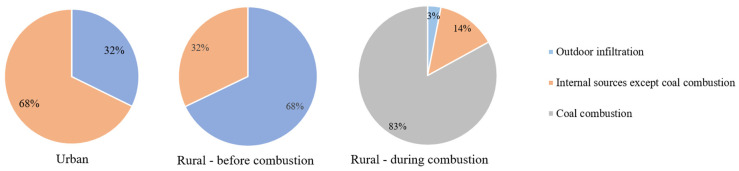
Contributions of outdoor infiltration, coal combustion, and other internal sources to the overall indoor UFPs with or without coal combustion.

Based on the simultaneously measured indoor and outdoor concentration over the study period, we further estimated the relative contributions of outdoor and internal sources to indoor UFPs. As seen in Figure 3, in the urban household without indoor coal combustion, outdoor infiltration was an important source of indoor UFPs, contributing to nearly 89.3% (IQR: 87.8–91.0%) of the indoor UFPs. In the rural household, outdoor UFPs contributed to about 67.9% (65.1–70.6%) of indoor UFPs during the period before coal combustion. When coals were burned indoors, a majority of UFPs were from the coal contribution, comprising up to nearly 82.8% (78.9–87.0%) of the indoor UFPs on average. The other internal sources and outdoor air contributed to 13.9% (10.5–17.2%) and 3.1% (2.5–3.9%), respectively. To our knowledge, there is still no quantitative estimation of source contributions to indoor UFPs. The statistical approach here provided a practical approach to identify the main sources and their contributions, although the results are rough. The sources of indoor PM_2.5_ and/or several gases have been studied in a few studies in the literature. Men et al. (2021) estimated the indoor heating source by burning coals contributed up to 47% of the indoor PM_2.5_ mass concentration during the heating period in rural China, while the other indoor sources (including cleaning and movement of people) contributed to ~20% of the indoor PM_2.5_ [47]. Lai et al. (2020) reported that the indoor biomass burning contribution to indoor PM_2.5_ ranged from 27% to 87% in rural Sichuan China, depending on the study location and season, as well as source apportionment methods [48]. These results, although varying in quantitative results, confirmed substantially high contributions of indoor combustion sources to indoor air quality.

### 3.4. Influence of Coal Combustion on PNSD (Particle Number Size Distribution) Indoors

**Figure 4:**
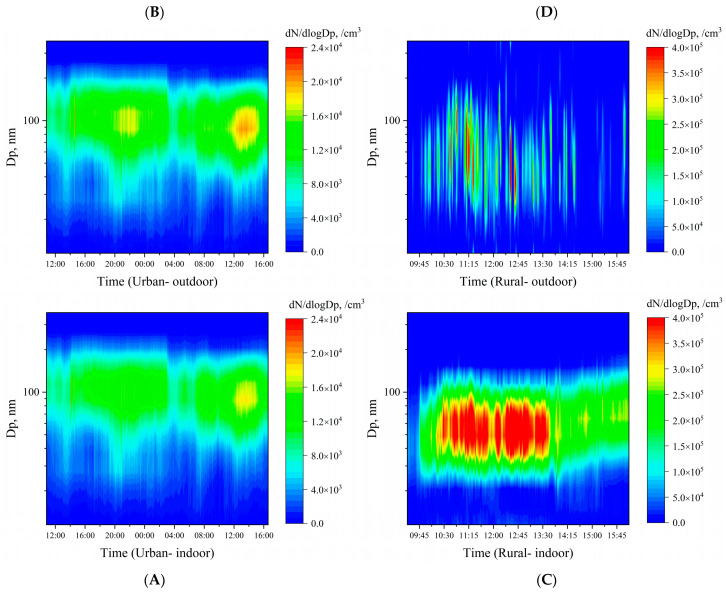
Dynamic changes and size distribution of UFP number concentrations in urban indoor (**A**) and outdoor (**B**), and rural indoor (**C**) and outdoor (**D**) households.

Being influenced by several factors, such as source emissions and meteorological conditions, particle number concentration and size distributions may change over time. The dynamic characteristics of UFPs with different sizes during the measurement period are illustrated in Figure 4. In the rural household with coal combustion, UFPs of 20–100 nm dramatically increased from the ignition to stable combustion periods, due to the high emissions of ultrafine particles in coal combustion [12,27]. During the stable combustion phase (after 14:00), though the total number concentration started to decrease, the overall median particle size slightly increased as small particles from the coal combustion became fewer.

In the urban room, ambient particle number concentrations reached a peak during the rush hour, especially for particles of about 100 nm. After the evening rush hour, the particle number concentration began to decline and reached the minimum at 3:00, which was consistent with observations by Buonanno et al. (2013) in public schools in Italy [45]. During the next day, particle number concentrations gradually increased because of emissions during the morning rush hours and stronger photochemical reactions under solar radiation [49,50,51,52]. Indoor UFP concentrations were significantly influenced by outdoor air and changed simultaneously. Particle size distribution had much fewer fluctuations in the urban household compared to the rural household.

## 4. Conclusions

UFPs have significant impacts on human health and regional climate. Studies on UFPs associated with solid-fuel use, especially those using coals, are relatively few compared to those on urban or public settings. In this study, we evaluated the indoor UFPs from a rural household burning coal for daily cooking and heating and the indoor UFPs from an urban home without indoor solid-fuel combustion as a comparison. Outdoor UFPs were measured simultaneously. Significantly high levels of UFPs in indoor air, especially with a coal-combustion source present, were found. The size distributions of UFPs in the indoor and corresponding outdoor air were similar, though the number concentrations were rather different. In the rural home, the indoor-to-outdoor ratio was about 6.4, confirming the strong impacts of indoor sources. With highly time-resolved data, the study estimated that indoor coal combustion can contribute to over 80% of indoor UFPs, while in the urban home without a strong internal source, the outdoor air contributed to about 90% of indoor UFPs.

The study is limited by a short study period and sample size, and it failed to evaluate inter-household variations and the influence of different factors (fuels, stove types, stove temperature, etc.). It would also be interesting to cover sub-micron and micron-size particles by using instruments such as APS (aerodynamic particle sizer spectrometer) and SMPS in future works. Indoor monitoring is usually more difficult than outdoor air studies, and it is affected by more factors, resulting in high inter- and intra-household variations. More studies call for a better understanding of the pollution status of UFPs in indoor air, and in support of UFP-related health studies and control policy development.

## Supplementary Materials

The following supporting information can be downloaded at: https://www.mdpi.com/article/10.3390/ijerph19095161/s1, Figure S1: The plans of the rural room (left) and the urban room (right) and the instruments’ locations. Figure S2: Frequency distribution of UFP number concentrations in outdoor (A) and indoor (B) environments without coal combustion indoors, and outdoor (C) and indoor air (D) with coal combustion source present.
