# Supplementary material for: Impacts of Household Coal Combustion on Indoor Ultrafine Particles—A Preliminary Case Study and Implication on Exposure Reduction
Source: Int J Environ Res Public Health. 2022 Apr 24;19(9):5161. doi: 10.3390/ijerph19095161 (PMC9101610; doi:10.3390/ijerph19095161)
Supplement: Supplementary file 1 [file ijerph-19-05161-s001.zip › ijerph-1655719-supplementary.pdf]

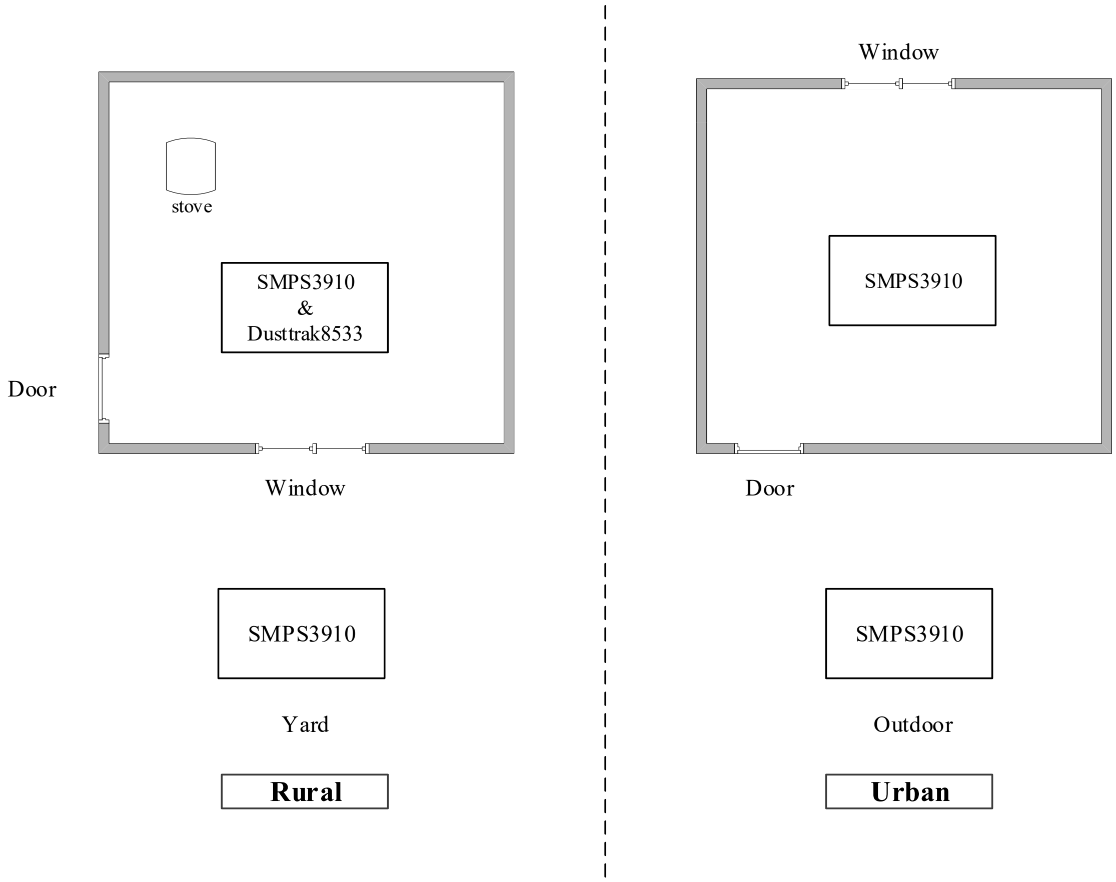

Figure S1. The plans of the rural room (**left**) and the urban room (**right**) and the instruments' locations.

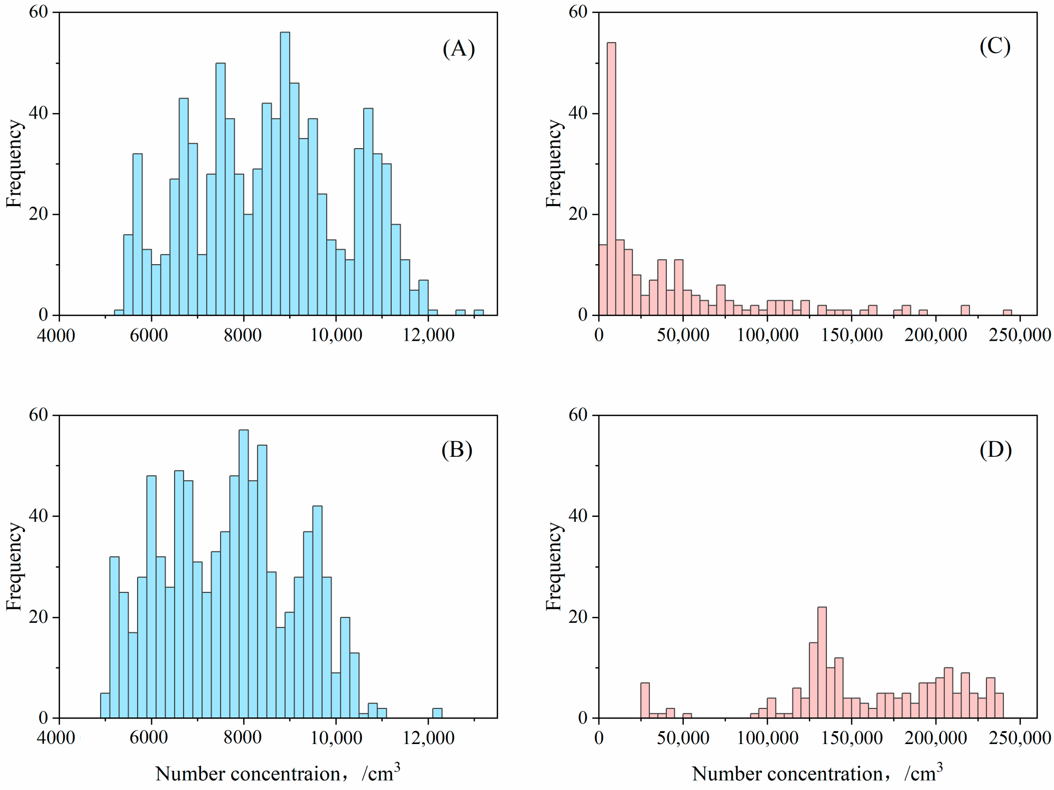

Figure S2. Frequency distribution of UFP number concentrations in outdoor (**A**) and indoor (**B**) environments without coal combustion indoors, and outdoor (**C**) and indoor air (**D**) with coal combustion source present.
